# Genomic approaches to identify hybrids and estimate admixture times in European wildcat populations

**DOI:** 10.1038/s41598-019-48002-w

**Published:** 2019-08-12

**Authors:** Federica Mattucci, Marco Galaverni, Leslie A. Lyons, Paulo C. Alves, Ettore Randi, Edoardo Velli, Luca Pagani, Romolo Caniglia

**Affiliations:** 1 Area per la Genetica della Conservazione (BIO-CGE), Istituto Superiore per la Protezione e la Ricerca Ambientale (ISPRA), Ozzano dell’Emilia, Italy; 2 Area Conservazione, WWF Italia, Rome, Italy; 3 Department of Veterinary Medicine and Surgery, College of Veterinary Medicine, University of Missouri, Columbia, USA; 4 Centro de Investigação em Biodiversidade e Recursos Genéticos (CIBIO), InBio - Laboratório Associado, Campus Agrário de Vairão, Vairão, Portugal; 5 Departamento de Biologia, Faculdade de Ciências, Universidade do Porto, Porto, Portugal; 6 Wildlife Biology Program, Department of Ecosystem and Conservation Sciences, University of Montana, Missoula, USA; 7 Department of Biological, Geological and Environmental Sciences, University of Bologna, Bologna, Italy; 8 Department of Chemistry and Bioscience, Faculty of Engineering and Science, University of Aalborg, Aalborg, Denmark; 9 Dipartimento di Biologia, Università degli Studi di Padova, Padua, Italy; 10 Estonian Biocentre, Institute of Genomics, University of Tartu, Tartu, Estonia

**Keywords:** Genetic hybridization, Conservation genomics

## Abstract

The survival of indigenous European wildcat (*Felis silvestris silvestris*) populations can be locally threatened by introgressive hybridization with free-ranging domestic cats. Identifying pure wildcats and investigating the ancestry of admixed individuals becomes thus a conservation priority. We analyzed 63k cat Single Nucleotide Polymorphisms (SNPs) with multivariate, Bayesian and gene-search tools to better evaluate admixture levels between domestic and wild cats collected in Europe, timing and ancestry proportions of their hybrids and backcrosses, and track the origin (wild or domestic) of the genomic blocks carried by admixed cats, also looking for possible deviations from neutrality in their inheritance patterns. Small domestic ancestry blocks were detected in the genomes of most admixed cats, which likely originated from hybridization events occurring from 6 to 22 generations in the past. We identified about 1,900 outlier coding genes with excess of wild or domestic ancestry compared to random expectations in the admixed individuals. More than 600 outlier genes were significantly enriched for Gene Ontology (GO) categories mainly related to social behavior, functional and metabolic adaptive processes (wild-like genes), involved in cognition and neural crest development (domestic-like genes), or associated with immune system functions and lipid metabolism (parental-like genes). These kinds of genomic ancestry analyses could be reliably applied to unravel the admixture dynamics in European wildcats, as well as in other hybridizing populations, in order to design more efficient conservation plans.

## Introduction

Anthropogenic hybridization, the cross-breeding of genetically differentiated *taxa* due to human alterations of habitats and populations, is one of the major threats to the conservation of native plants and animals^1–3^. Hybridization between free-ranging domestic animals and their wild conspecifics may spread artificially-selected maladaptive variants causing fitness declines, outbreeding depression and gradual alterations of locally adapted gene complexes, thus increasing the risk of extinction of wild populations or entire species^2,4–8^. However, recent studies documented cases of beneficial introgression of domestic mutations in wild populations of North American wolves (a melanic deletion at the β-defensin-103 locus^9^) and Alpine ibex (a domestic goat MHC haplotype^10^).

Cross-breeding between wild and domestic cats, intensified by the human-mediated worldwide dispersal of domestic cats (*Felis silvestris catus*), together with the demographic decline and fragmentation of European wildcat populations (*F*. *s*. *silvestris*^11^), offers a remarkable case-study of anthropogenic hybridization^12,13^. The widespread diffusion of stray or feral cat populations^14^ likely promoted reproductive interaction between the two subspecies. Moreover, the full fertility of their hybrid offspring^15^ due to the recent origin of the domesticated cat^16,17^ has likely increased the risk of genetic introgression. Despite the active role of ecological barriers found to limit hybridization in some Mediterranean regions^18,19^, variable degrees of admixture have been detected throughout the European continent and across habitat types as a consequence of human pressures^2,20–26^, leading to a complete hybrid swarm in wild-living cats in Scotland^12^. Such geographical heterogeneity in admixture levels might be explained by different environmental conditions and ecological barriers^19^, population histories and proportions^20,27^, or by the choice of markers and sampling design^26^.

However, the fitness consequences of introgressive hybridization in wildcats are still unknown. In other species, specific genes or gene complexes of domestic origin could either show selective advantages or, in contrast, reduce fitness or induce outbreeding depression^4,7,28^. Therefore, the accurate detection of hybrids, the quantification of introgression in hybridizing populations and the identification of their demographic and ecological determinants are needed for developing appropriate wildcat conservation plans and correctly allocate resources for their application^29,30^.

Variations in coat color patterns and morphological traits between wildcats and domestic cats are not always diagnostic^31,32^, thus their hybrids and backcrosses are not easily identifiable through the analysis of morphological features. Hence, hybridization has been more reliably assessed using molecular markers, mainly small panels of hypervariable microsatellites (short tandem repeats, STRs) and short mitochondrial DNA (mtDNA) sequences. The high variability of these markers, analyzed using Bayesian and phylogenetic statistical tools, has radically improved our knowledge of the European wildcat population genetic structure^20,24,27,33–37^, but showed a limited power to investigate the ancestry of admixed individuals. The wild and domestic cats used as parental references for multivariate and Bayesian assignment analyses were often regionally sampled, and the different applied marker panels were seldom comparable^12,13,21–24,31,32,34,38,39^. Consequently, standardized and more powerful panels of molecular markers to be applied at a large scale are required to lower the risk of underestimating the prevalence of introgressive hybridization in natural wildcat populations.

Recent next-generation sequencing platforms can offer solutions allowing the assemblage of extensive and cost-effective panels of ancestry-informative markers (AIMs) constituted by Single Nucleotide Polymorphisms (SNPs), which represent the most widespread source of genome-wide variation^40^.

A promising set of 96 nuclear and mitochondrial SNPs has been recently selected by Nussberger *et al*.^25^ because of their fixed allelic differences between domestic and wild reference cats and applied for European wildcat admixture analyses. Furthermore, an increased set of SNPs (n = 158) has been identified by Oliveira *et al*.^30^ from the 1.9 × genome sequence of an Abyssinian domestic cat^41^ because of their informativeness and variability in domestic cat breeds^42^. Both marker panels were applied on a wide cat sampling and proved to be able to successfully assign hybrid categories up to the second admixture generation (with a mean error rate value of 2–12% in category assignment^26,30^). However, these SNPs showed a limited power to detect old-generation backcrosses resulting from a repeated cross-breeding between admixed individuals and parental species.

Recent studies showed how the employment of thousands of markers might help to reveal previously undetectable backcrosses (older than two–three generations in the past) and estimate the timing from the admixture events^43,44^. Additionally, the availability of efficient AIMs widely distributed across the entire genome can allow to identify patterns of introgressed linkage blocks hosting candidate genes that may underlie introgressed functional traits that are still unknown^45,46^, further help to disentangle historical and contemporary admixture^47^ by analyzing the distribution of haplotype block lengths^46,48^ and associate anomalous phenotypes with their genetic bases^44^. These approaches allow researchers to better understand the dynamics and consequences of anthropogenic hybridization compared to previous studies, helping to face specific management and conservation issues^46^.

The recently released Illumina Infinium iSelect 63k DNA cat array contains 62,897 variants that are mostly polymorphic within the domestic cats and includes 4,240 wildcat-specific markers^49^. This array offers a suitable molecular tool to further investigate the ancestry of European wildcat populations in conservation and monitoring projects^49^.

Here we genotyped a wide sampling of European wildcats, domestic cats and known or putative admixed cats from a large part of the European wildcat home range distribution with the Illumina Infinium iSelect 63k DNA cat array by applying multivariate, Bayesian and gene-search analysis tools to: (1) improve the identification of admixed genotypes older than the first few generations of backcrossing, (2) estimate their times of admixture^50–52^, (3) quantify and localize domestic and wildcat-derived genomic regions, (4) search for genes significantly deviating from random inheritance patterns, possibly due to selective pressures, (5) define a reduced panel of AIMs to routinely apply for population structure and hybridization monitoring projects.

## Results

### Data filtering and marker selection

Quality-control and filtering procedures yielded a final sample set consisting of 80 presumed European wildcats (WC), 44 domestic cats (DC) and 22 known or presumed WC x DC admixed individuals (Supplementary Fig. S1), previously identified from STR assignment and multivariate analyses^20,21,27,37^, successfully typed for 57,302 autosomal SNPs, hereafter referred to as the 57k SNP panel set (35,228 after linkage disequilibrium pruning, hereafter referred to as the 35k LD-pruned SNP panel set).

### Assignment and admixture analyses of the sampled cats

**Figure 1:**
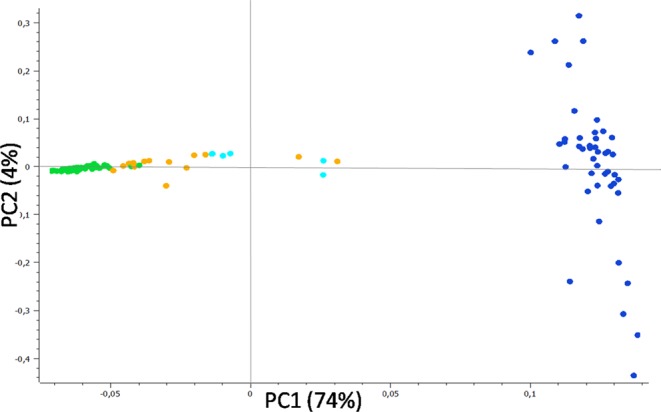
PC1 *versus* PC2 results from an exploratory principal component analysis (PCA) computed in SVS on the 57k SNP panel set and including domestic cats (blue dots), putatively admixed wildcats (orange dots), known captive hybrids previously genetically identified with STR data (light blue dots) and European wildcats (green dots). The two axes are not to scale, in order to better distinguish individuals along PC2.

More than 78% of the genetic variability of the sampled cats was explained by the first two components of a preliminary Principal Component Analysis (PCA) performed in SVS using the 57k SNP panel set (Fig. 1), which clearly distinguished domestic from wildcats. Putative admixed cats (referred to hybrids and backcrosses), genetically identified through previous STR analyses^20,21,27,37^, were scattered along the first axis (74%) between the parental cats, mainly closer to the wildcat group, except for the known hybrids (referred to F1-F2 individuals), which were intermediate (Fig. 1).

**Figure 2:**
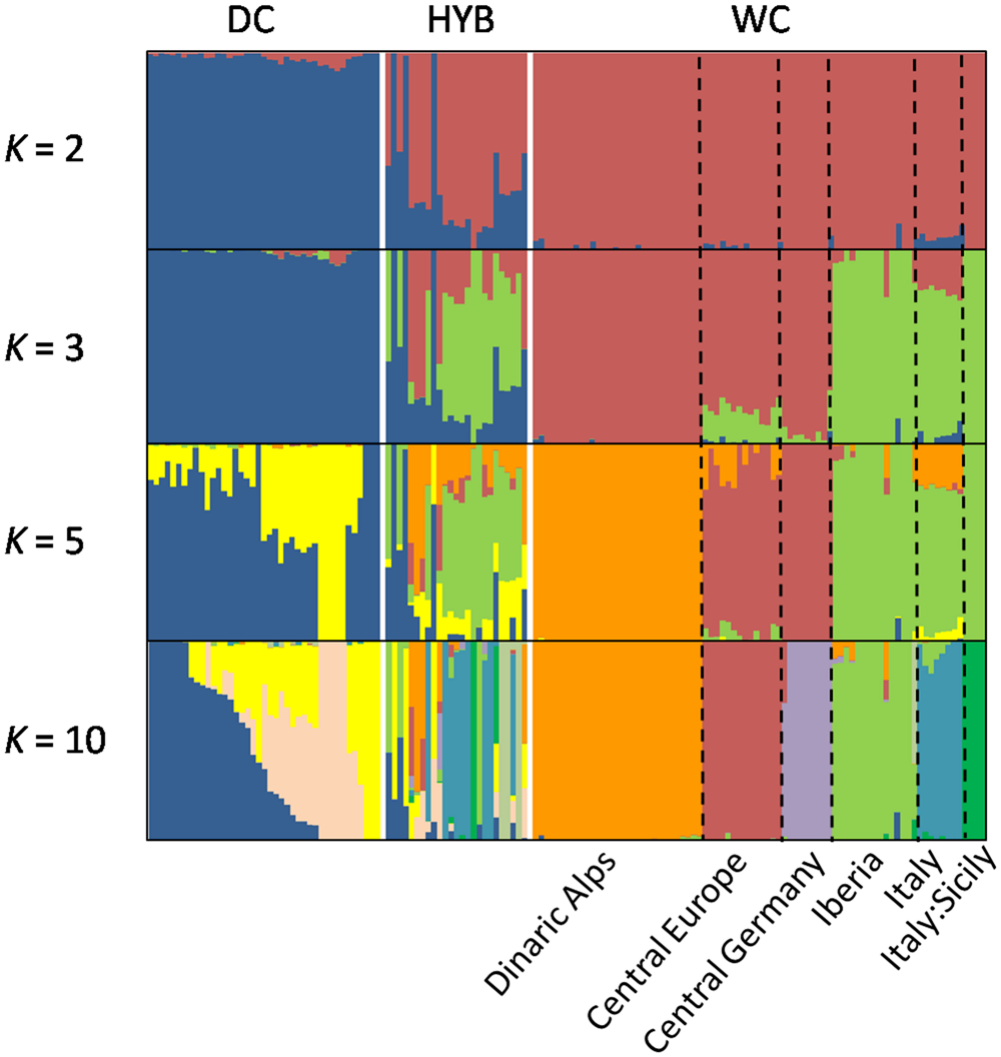
Admixture results from the 35k LD-pruned SNP panel set at K = 2, K = 3, K = 5 and K = 10. K = 2 clearly separates wild from domestic cats with admixed individuals showing intermediated assignment values. From K > 2 the genetic substructure of European wildcats progressively takes shape. At K = 3 the Northern European wildcat populations (including the Dinaric, the Central European and the Central Germany areas) split from the Southern ones (including the Italian and the Iberian Peninsulas). At K = 5 the Dinaric wildcat population groups apart from the Central European and the Southern ones and domestic cats form two distinct sub-clusters. At K = 10 the five biogeographic macro-populations already identified in Europe through STR analyses (Iberian, Italian, Central European, Dinaric and Central Germany populations^27^) are confirmed, with an additional cluster including the Sicilian samples.

Multivariate analyses were clearly confirmed by the assignment values obtained from the Admixture tests performed with the 35k LD-pruned SNP panel set that showed the main decrease in the cross validation error (CV) at the optimal genetic clusters K = 2 (Supplementary Fig. S2) and clearly separated domestic cats from wildcats (Fig. 2). Based on the distribution of individual assignment values, we preliminarily identified the parental reference cats and the admixed individuals that we further investigated in the subsequent ancestry analyses. All domestic cats (n = 44) showed an individual assignment value q_w_ < 0.090 and were considered as reference domestic sources, whereas for the putative wildcats, whose individual membership values q_w_ ranged from 0.870 to 1.000, we defined a strictly conservative q-threshold that retained as reference wild sources only individuals with a q_w_ = 1.000 (n = 57). Therefore, we considered as admixed all cats showing an intermediate assignment value 0.090 > q_w_ < 1.000 (n = 45). For K > 2, the genetic substructure of European wildcats^31^ progressively took shape, with the initial split of the southern European wildcat populations (Italian and Iberian Peninsulas) from the Central-Northern cats (including the Dinaric and the Central European areas) observed at K = 3 (Fig. 2), followed by the subsequent isolation of the Dinaric population at K = 5 (Fig. 2), and by the final split of five main biogeographic clusters (Iberian, Italian, Central European, Dinaric and Central Germany populations) as previously identified in Europe^27^. An additional cluster represented by Sicilian samples was identified at K = 10 (Fig. 2).

A highly significant admixture rate between domestic cats and European wildcats was corroborated by the ThreePOP results for the F3 tests^53^ computed in all the putative admixed individuals detected with Admixture (z-score = −113.04) and confirmed by analyzing the five European wildcat macro-areas separately (z-scores ranging from −8.22 of the Central Germany to −35.70 of the Central Europe, from −113.11 and −49.19 of the Iberian and Italian Peninsulas to −91.78 of the Dinaric macro-area).

The observed widespread signals of genomic admixture were individually estimated with PCAdmix which identified an average 17% of domestic regions (with only slight variations across chromosomes, ranging from 16% in Chr5 to 19% in Chr9, see Supplementary Fig. S3) in the genome of the putative admixed cats. The proportions of domestic blocks within individuals ranged from 1.8% to 65.3%, significantly correlated (R^2^ ≥ 0.95; P-values = 1.72 × 10^−9^; t-test) with those estimated in Admixture at K = 2 (mean q_d_ = 0.141). None of the 40 randomly-selected reference individuals reanalyzed as hybrids for comparison in PCAdmix showed any switch from domestic to wildcat blocks (or *viceversa*) along their genomes, confirming the reliability of the reference populations selected for the admixture timing analyses and the possibility to exclude any ascertainment bias from the SNP array.

### Time of admixture

We inferred the time in generations during which the admixture events between domestic and European wildcat populations took place by analyzing patterns of linkage disequilibrium decay^54^ in Alder. Significant admixture was detected in the five European wildcat biogeographic areas identified with Admixture (P-values < 3.5 × 10^−8^), although with inconsistent decay rates in all the cohorts considered, except for the Dinaric macro-area. The admixture midpoint in the European wildcats was generally estimated in Alder to have occurred about 5.02+/−0.37 generations before sampling, which correspond to about ten years considering a cat generation time of two years. The most ancient hybridization events were detected in the Italian Peninsula (6.62+/−0.58 generations) and in the Central European area (8.60+/−1.56 generations), respectively corresponding to about 14 and 18 years before sampling, whereas a more recent admixture time of about six years was estimated in the Dinaric region (3.15+/−0.24 generations).

**Figure 3:**
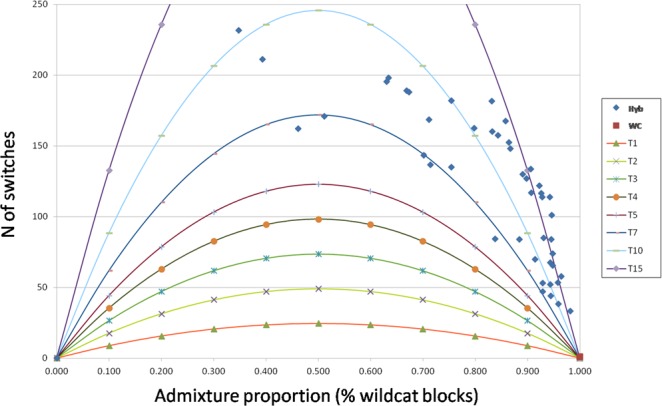
Timing since the admixture event for each admixed individual (Hyb = 45), deduced from the empirical distribution of the number of chromosomal switches inferred from PCAdmix, in relation to the individual assignment values (proportion of wildcat blocks). The colored lines indicate the expected distributions at increasing generations since admixture.

The local ancestry inferred with PCAdmix in single individuals identified a number of switches from the reference European wildcat ancestry blocks to domestic cat blocks ranging from 31 to 231 (mean value 122 ± 9), revealing that all the admixture events within the European wildcats occurred at least six generations before sampling (Fig. 3 and Supplementary Fig. S4), with the oldest timing estimated up to 22 generations before sampling. Coherently with Alder, the most ancient hybridization events were traced in the Italian Peninsula (mean generation value 13 ± 9), while the most recent events in the Dinaric Alpine populations (mean generation value 8 ± 9). This pattern dated the first case of hybridization in the Italian Peninsula to 1962 (corresponding to 44 years before sampling), whereas the last admixture event likely dated to 1994 (*ca*. 14 years before sampling) in the Dinaric region (Supplementary Fig. S4). Although significantly (P-values = 2.69 × 10^−3^; t-test) correlated, the average admixture timing estimated with PCAdmix resulted approximately twofold more ancient compared to the midpoints estimated by Alder.

### Regions of genomic differentiation between domestic and European wildcats

The admixed cats revealed a complex genomic mosaic of wild and domestic ancestry, as reconstructed by PCAdmix. Therefore, 138 regions with high frequency of wildcat alleles (wildcat-like regions) were identified, including 1,045 annotated genes, 577 of which were significantly enriched for specific Gene Ontology (GO) categories and are known to be involved in several biological and cognitive processes related to communication and elusive behaviors (Table 1 and Supplementary Table S1c,d). In particular, we observed genes belonging to significantly enriched Cellular Component (CC) categories playing important roles in memory performance and sociability, or being related to development processes, key morphological features and fertility (Table 1 and Supplementary Text S1).

**Table 1:** Subset of significantly enriched GO domestic-like (a) and wildcat-like (b) outlier genes detected in the domestic *x* wildcat admixed cats, identified with Bayesian analysis in Admixture and PCAdmix, which have been previously described in literature.

Ancestry	Association	Gene name
FCA-like	Fear of human	*GPRC5A* ^85^
	Stress responses	*CAPZA1* ^86^
	Neural crest development	*CTNNB1* ^87, 88^
	Tumor suppressor	*PHLDB2*^89^; *TRIM29*^90^; *PTPRT*^91^
	Immune system (IAV severity)	*ZC3HAV1* ^92^
	Immune system (H5N1 virus)	*SLC16A1* ^93^
	Lipid metabolism	*PLPP3* ^94^
FSI-like	Memory performance	*ADCY8*^95^; *CUX2*^96^
	Fear of human	*DYNLRB2*^85^; *MRPS18A*^97^
	Tameness and aggressiveness	*SLC6A4*^98^; *SPTLC3*^99^
	Sociability	*MAD2L1BP*^100^; *ATP8A1*^101^; *ADCY7*^102^
	Brain development	*DCTN2* ^103^
	Brain development, white coats pattern	*KIT* ^104, 105^
	Development processes	*EDN1*^106^; *KIF2B*^107^; *BHMT*^108^;
	Development processes (milk yielding)	*ACP6*^109^; *ARHGEF4*^110^; *MTX3*^110^
	Fertility (spermatogenesis regulation)	*MORN3*^111^; *NKD1*^112^
	Fertility (maintenance pregnancy)	*MCL1*^113^; *LNPEP*^114^
	Fertility (likelihood of survival)	*RNPEP* ^115^
	Morphology	*NPR2*^116^; *PIK3R4*^117^; *STX17*^118^
	Muscle development	*MYLPF*^119^; *COX6A2*^120,121^
	Tumor suppressor	*BIN1*^122^; *CUL9*^123^; *GSDMC*^124^ *NOL7*^125^
	Immune system (FeLV virus)	*TRIM25* ^126^
	Immune system (ASFV virus)	*NFKBIE* ^127^
	DNA repair	*CMPK1*^128^; *DDIT3*^129^; *CCDC92*^130^
	Lipid metabolism	*ALDH2* ^131^
	Disease	*BCL9*^132^; *BFSP2*^133^
	Disease (Feline Spinal Muscular Atrophy)	*LIX1* ^134^
	Disease (Hypertrophic Cardiomyopathy Mutations)	*MYL2* ^135^
	Disease (hearing disorders)	*MYO1A* ^136^

Moreover, 138 segments with high frequency of domestic alleles were identified, containing 902 annotated genes, 39 of which were significantly enriched for Human Phenotype (HP) and Molecular Function (MF) categories correlated to cell adhesion molecular binding (crucial for maintaining tissue structure and function; Supplementary Table S1a,b). Interestingly, we found domestic-like genes significantly enriched in GO categories mainly associated with neural crest development cognition and behavior, or related to biological immune system responses and physiological adaptations (Table 1 and Supplementary Text S1).

Both wild- and domestic-like regions hosted a number of significantly enriched GO genes implicated in muscle development, lipid and energy metabolism or known to be involved in immune functions, tumor suppressor and DNA repair functions (Table 1 and Supplementary Tesxt S1). Another set of wild- and domestic-like enriched GO genes were described as associated with diseases or infections, some of which were feline-specific (Table 1 and Supplementary Text S1).

Conversely, none of the F_ST_ outlier SNPs showed a significantly positive P-value in BayeScan, suggesting no evidence of selection signatures neither comparing admixed individuals *versus* wildcats nor comparing admixed individuals *versus* domestic cats.

### Selection of informative SNPs

A reduced panel of SNPs was selected based on estimates of WC-DC divergence (F_ST_ and I_N_) that were highly correlated one another (Spearman’s r F_ST_ – I_N_ = 0.99; P < 0.0005), and even to the H_E_ values (differences not significant at X^2^ = 1; P > 0.25 Chi-test; Supplementary Fig. S5). Thus, for instance, SNPs showing the lowest H_E_ values (0.010) had also the lowest average F_ST_ (0.002) and average I_N_ (0.003) values. Based on these results, we selected the top 192, 96 and 48 SNPs showing the highest F_ST_ values and evaluated their performance using a PCA visual summary of their observed genetic variation.

**Figure 4:**
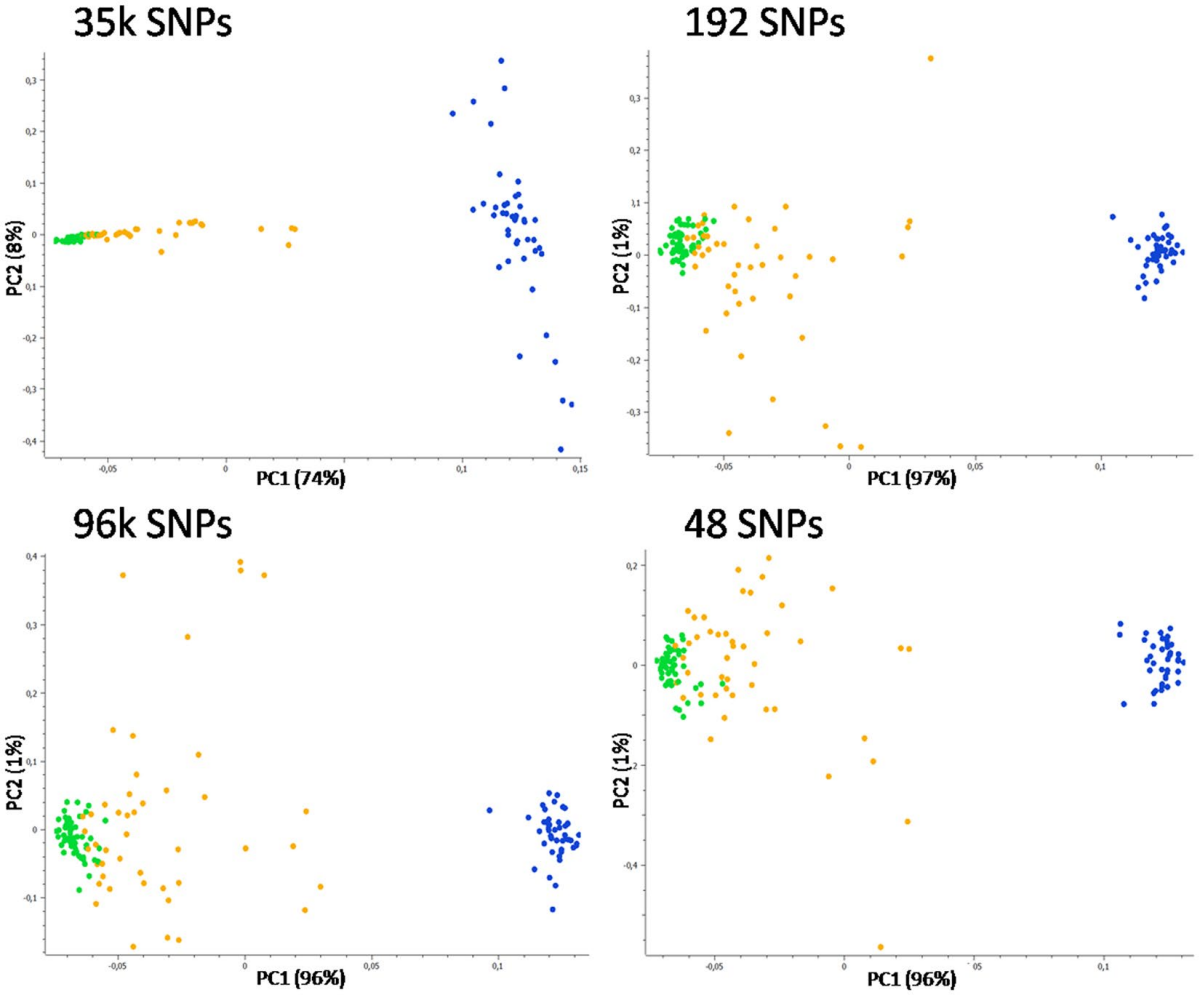
Principal component analysis (PCA) computed in SVS on the 35k LD-pruned SNP panel set and on the 192, 96, 48 SNPs showing the highest wild-domestic cats F_ST_ and I_N_ values. For each dataset, PC1 *versus* PC2 are indicated (axes are not to scale). Domestic and European wildcats are represented in green and blue dots, respectively, known hybrids and putatively admixed wildcats (Admixture q_w_ < 0.999) in orange. The power of the top 48 SNPs is comparable to that reached with 35k SNPs, indicating that they can be used as reliable ancestry-informative-markers (AIMs), although no clear subdivision could be traced between some of the admixed and the non-admixed wildcats.

Results were highly concordant and all the reduced panels, which included from 6 to 23 wildcat-specific variants previously described in Gandolfi *et al*.^49^, well differentiated domestic cats and wildcats (192 top SNPs: F_ST_ = 0.90, H_O-WC_ = 0.060, H_O-DC_ = 0.092; 96 top SNPs: F_ST_ = 0.93, H_O-WC_ = 0.074, H_O-DC_ = 0.039; 48 top SNPs: F_ST_ = 0.95, H_O-WC_ = 0.029, H_O-DC_ = 0.057), grouping most admixed individuals intermediately, with the exceptions of about ten genotypes that plotted more closely to the wildcat group (Fig. 4). The assignment values from the Admixture run at K = 2 on the 192, 96, and 48 SNPs were not significantly different from values obtained with the 35k LD-pruned SNP panel set (P-values > 0.5 in all cases; t-test), although a portion of putative admixed cats (q_w_ < 1.000 in PCAdmix), ranging from 9% to 22% (with the 192 and 48 SNP panel sets, respectively), were misclassified and confused as parental wildcats (Table 2).

**Table 2:** Performance of the five reduced SNP panel sets in assignment procedures performed in Admixture on wildcat (n = 57), domestic (n = 44) and admixed individuals (n = 45), previously identified using the 35k LD-pruned SNP panel set in clustering analyses (see Results).

	192 SNPs	96 SNPs	48 SNPs	96 + 96 SNPs	48 + 48 SNPs
WC Average q_w_ value	0.990	0.991	0.988	0.991	0.989
WC min q_w_ value	0.953	0.948	0.936	0.949	0.944
WC misclassified	32 WC q_w_ < 1.000	28 WC q_w_ < 1.000	28 WC q_w_ < 1.000	27 WC q_w_ < 1.000	26 WC q_w_ < 1.000
Hyb misclassified	4 Hyb q_w_ > 0.953	6 Hyb q_w_ > 0.948	10 Hyb q_w_ > 0.936	7 Hyb q_w_ > 0.949	10 Hyb q_w_ > 0.944
% Hyb identified	9	13	22	16	22
Hyb generation	8–19	8–19	8–19	8–19	8–19
Hyb PCAdmix q_w_ value	0.929–0.982	0.929–0.982	0.929–0.982	0.929–0.982	0.929–0.982

Average and minimum membership proportions to the wild cluster of the reference wildcats (WC), number of misclassified WC showing assignment values q_w_ < 1.000 compared to the 35k-LD pruned SNP panel set results (q_w_ = 1.000), number of misclassified admixed cats (Hyb) showing assignment values q_w_ ≥ minimum WC q_w_, percentage of hybrids correctly detected (assignment values q_w_ < minimum WC q_w_), generation time and individual membership proportions of admixed individuals retained by PCAdmix analyses on the 35k LD-pruned SNP panel set, are shown for each AIM SNP panel set.

The genetic variability of the five main bio-geographic wildcat groups, summarized using the top 96 (F_ST_ = 0.93, H_O_ = 0.103) and 48 (F_ST_ = 0.93, H_O_ = 0.077) informative SNPs, selected based on WC divergence (F_ST_) and graphically plotted in a PCA (Supplementary Fig. S6), was concordant with the Admixture results previously described (Fig. 2).

**Figure 5:**
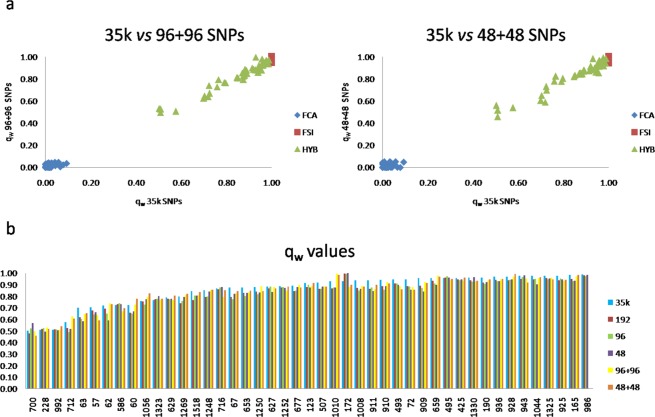
(**a**) Scatterplot of individual proportions of membership to the wild clusters (q_w_) of 146 sampled cats (including domestic, European wild and known/putatively admixed cats), according to the assignment analyses performed in Admixture. Individual’s wild memberships estimated with the combined reduced SNP panel set (96 + 96 and 48 + 48 AIM SNPs, informative for both admixture and genetic structure analyses) were strictly correlated with those obtained with the 35k LD-pruned SNP panel set. (**b**) Wildcat ancestry proportions of known hybrids and putative admixed cats (n = 45) inferred in Admixture with the initial 35k LD-pruned SNP panel set in addition to the reduced (192, 96 and 48) and combined (96 + 96 and 48 + 48) SNP panel sets. All the reduced marker panels did not reduce the assignment power to the parental clusters.

The combined panel of SNPs, selected based on both WC-DC and WC divergence (F_ST_), mostly confirmed Admixture results (Table 2). However, 16% (using 96 + 96 top SNPs, PID_WC_ = 5.6 × 10^−33^; PIDsib_WC_ = 3.7 × 10^−17^) and 22% (using 48 + 48 top SNPs, PID_WC_ = 1.1 × 10^−14^; PIDsib_WC_ = 8.2 × 10^−8^) putative admixed individuals were misassigned and confused as reference wildcats, despite their high PCAdmix q_w_ values ranging from 0.929 to 0.982 (Table 2) and their ancient origin estimated from 8 to 19 generations before sampling (Table 2). Interestingly, the combined SNP panel sets did not reduce the assignment power to the parental clusters and admixed qi values were strictly correlated with those obtained from the 35k LD-pruned SNP panel set (R^2^ = 0.971; P < 0.0001 for 35k − 96 + 96 SNPs; and R^2^ = 0.962; P < 0.0001 considering 35 k − 48 + 48 SNPs), see Fig. 5.

## Discussion

Human-mediated processes, such as habitat destruction, human persecution and anthropogenic hybridization, can directly or indirectly threat global biodiversity because of their unpredictable consequences on the fitness of natural populations^4,7^. In this study, thanks to the availability of a well-annotated reference genome (FelCat8 – *Felis_catus_*8.0^55^), we performed a genome-wide assessment of admixture patterns and timing in a number of European wildcat populations. A preliminary genomic screening, based on pairwise F_ST_ values, multivariate and assignment procedures, showed that wild and domestic cats remain highly differentiated and well-distinguished. All the analyzed putative admixed cats confirmed to bear admixture signals and were well-identifiable, though some of them were very close to the wildcat group, ranging from *c*. 50% domestic-derived ancestry to almost complete wildcat assignments. Considering a neutral perspective, these patterns clearly indicate that ~40% of the analyzed admixed individuals would fell within the first three hybrid generations, whereas ~60% could represent more ancient backcrosses in which the domestic legacy would have been diluted through time. Genome-wide assignment procedures were also highly efficient in detecting population substructure, since five main biogeographic European wildcat macro-populations were clearly identified, consistent with previous findings based on data generated using 31 autosomal microsatellite loci^27^.

PCAdmix results showed that admixed animals mostly originated from 7 to 14 generations in the past, with some individuals older than the twentieth generation of backcrossing, thus detecting hybridization events occurred between 1962 and 1994 considering a generation time of two years. In particular, the most ancient admixture traces were detected in individuals which had been misclassified as pure in previous microsatellite-based analyses, confirming the deeper diagnostic power of genomic data in detecting past backcrossing events^56–58^. Alder results estimated the midpoint of hybridization at approximately five generations in the past, compared to the mean value of 12 generations extrapolated from ancestry switches. Such discrepancies between these admixture-dating methods might be attributable to PCAdmix algorithms that are more efficient in detecting more ancient hybridization traces in the genome of the hybrids by identifying their residual domestic blocks. Conversely, Alder algorithms are more suitable to identify the major admixture event (if a main one occurred), the midpoint (in case of continuous admixture events) or the latest events (if these were punctuated). Such timing patterns, even if preliminary, can provide additional information about the context and the period during which hybridization occurred in our analyzed samples, and thus can be useful to better understand causes and dynamics of the phenomenon at local scale. However, we cannot exclude that more ancient hybridization events have remained undetected in the European cat populations we analyzed since (1) the 63k SNPs still offer a moderate snapshot on the whole *Felis* genome, (2) our sampling design was not homogeneous neither in time nor in space, and (3) data about the collection year were not available for all the analyzed individuals. Future analyses based on a wider sampling and the comparison of entire genomes might shed more light on patterns and histories of admixture.

Interestingly, the six known hybrids, coherently with their possible F2 origins declared in previous analyses^20,21^, had assignment values ranging from 0.347 to 0.673, although they actually showed admixture traces dating back from 9 to 11 generations in the past. Such findings would suggest these animals might represent the product of repeated crosses among F1 or F2 individuals, rather than true second generation hybrids, whose domestic components could be detected only through the haplotype block analyses. These results highlight the power of such methods in improving the assessment of admixture proportions and timing from genomic data by detecting domestic-like and wild-like local genome ancestry better than the assignment procedures or morphology alone^58,59^.

The employment of thousands of SNPs, in fact, allowed us to distinguish backcrossed individuals with small proportions of domestic genome introgressed from wildcat parental populations, by accounting for the number of generations since secondary contact, which can occur when two (or more) species that have been in allopatry come back into sympatry.

Even if some studies hypothesized that ecological barriers can play a key role in limiting hybridization in some areas (as occurred in some Mediterranean regions^18,19^), variable levels of admixture were detected in all the sampling pools representative of five European wildcat macro-populations we analyzed. Such evidence suggests that, in absence of strong ecological barriers, hybridization can potentially threat some of the extant wildcat populations, including those living in the Italian Peninsula, as previously described not only for the wildcat^37^ but also for other mammalian species such as the wolf^44^, the roe deer^60^ and the wild boar^61^.

Since the samples we used in our analyses were not randomly selected, but mostly based on their DNA quality, and included animals collected from previously known areas of suspected hybridization^27,30^, this study did not allow to estimate hybrid prevalence nor the origin and spread of introgression in the local European wild cat populations.

Therefore, we suggest that dynamics and prevalence of hybridization in the European wildcat populations should be better estimated (1) through extensive country-wide sampling programs and genetic analyses of wounded or found-dead wildcats and (2) by well-planned local intensive non-invasive genetic and camera-trapping monitoring projects in hot spots of known or suspected hybridization throughout the entire wildcat distribution range. This approach should avoid the risk that carcasses of introgressed individuals might be confused with feral domestic cats and thus not analyzed, and it would allow to simultaneously obtain detailed phenotypical and genetic information at a European scale, as well as potential capture-recapture data to provide more reliable estimates of population size and hybrid prevalence.

The reduced panels of AIMs we selected from the Illumina Infinium iSelect 63k DNA cat array, based on multiple criteria of genetic differentiation and linkage independence, appeared to be particularly suitable for future large-scale monitoring projects in territories with high conservation priority and in areas of supposed or documented hybridization since (1) they allowed to distinguish individuals, even strictly related, without ambiguity (probabilities of identity <0.001), (2) clearly identified the geographic and genetic European wildcat macro-population structure and variability, (3) were highly concordant with the 35k LD-pruned SNP panel set and more accurate than the small number of previously used microsatellite loci in the identification of admixed individuals.

These reduced SNP panel sets could be easily integrated with other AIMs previously identified in cat admixture studies^25,30^ and with markers that will hopefully emerge from coordinated ongoing genomic studies^26^. Routinely, SNP genotyping of both invasively and non-invasively collected samples could be carried out through innovative analysis methods such as quantitative PCR^62^ or microfluidic^63–66^ techniques, which allow the cost-effective genome-wide characterization of dozens of samples and markers at a time, even starting from low DNA quality or quantity materials. Such approaches turned out to be highly reliable both for multilocus DNA fingerprinting reconstructions and for the correct identification of admixed individuals until the second backcross generation for a number of *taxa* such as the brown bear^64,66^, the wolf^62,63^ and the wildcat^25,26,65^.

We also capitalized the availability of the domestic cat SNP array dataset remapped on the *Felis_catus_*8.0 genome assembly^49^ to search for specific genes hosted in both domestic and wildcat-inherited regions, which might be associated with specific biological and phenotypical ontology processes as adaptive response to selective pressures.

The significantly enriched CC wildcat-like genes we identified in the admixed cats were mainly related to brain development and cognitive processes which also regulate aggressiveness and elusive behaviors typical of wild species. A variety of wildcat-like genes included in enriched CC categories were further found to be related with morphological features (such as genes regulating body size and hair coats) and development processes (such as muscle development and the energy metabolism), which might influence the body growth and composition resulting from adaptive pressures. Interestingly, we also identified a few significantly enriched CC genes correlated with fertility (*MORN3*, *NKD1*), the maintenance of pregnancy (*MCL1*, *LNPEP*) and the likelihood of survival (*RNPEP*), that might contribute to increase the fitness of the admixed individuals living sympatrically with wildcats.

Nonetheless, we also found four domestic-derived genes significantly enriched for GO categories mostly related to cognition and behavior, physiological adaptations and neural crest development, whose cellular deficit during embryonic development has been demonstrated to directly or indirectly modify several morphological and physiological traits, as well as to influence tameness during cat domestication, in agreement with the domestication syndrome hypothesis^67^. Such genes might have been maintained in the genome of the introgressed cats thanks to their possible adaptive roles in human-dominated landscapes, where a number of variables are highly modified by the human presence and actions (high density of domesticated *taxa* and their pathogens, habitat fragmentation and perturbation, modified circadian rhythms of prey, etc.) or even in quasi-natural contexts, as demonstrated for the domestic goat MHC haplotypes in the Alpine ibex^10^ and the dog-derived black coloration in wolves^9^. The possibility that similar patterns result from an ascertainment bias linked to the original SNP chip design, mostly based on the domestic cat variation, is very unlikely for closely related *taxa* diverging less than one million years^68^.

Cats have experienced a self-domestication history^69,70^ in which strong pressures operated by breeding strategies selecting for specific physical features occurred only recently and with limited effects on behavioral traits. Therefore, their gene pool has been poorly isolated from their wild counterparts, and the number of genomic regions with strong signals of selection and differentiation since cat domestication appeared modest^71^ compared with those reported in another domesticated species, the domestic dog^72^.

However, though gene enrichment analyses can provide a broad sense of the type of functions that are common to a significant number of genes (in this case, the ones hosted in regions found to be outlier for domestic or wild ancestry), our gene-search approach only allowed us to gain a preliminary insight on the inheritance patterns of domestic and wild ancestry blocks. Indeed, no significant evidences of selection signatures were detected by tests based on F_ST_ outliers, which are a more direct estimate of deviations from neutrality at a given marker. Such lack of selection could rely on the limited samples analyzed, or reflect the actual absence of differential selection for wild-derived or domestic-derived alleles in the admixed individuals. Therefore, all these data will need to be integrated in the future with systematic studies on fitness of the admixed individuals, including survival and breeding rates, in order to better understand the adaptive patterns of wild-living admixed individuals.

In conclusion, in this study we provide a comprehensive genome-wide approach to detect the occurrence and infer the timing of admixture events in the European wildcat populations investigated, improving the reliability of old-generation backcross identification and pinpointing a number of outlier genes possibly influenced by natural and artificial selection in samples collected from the main genetic macro-populations in Europe^27^. On average 17% of domestic ancestry were detected in the genomes of most analyzed putative admixed cats, which were all classified as backcrosses more ancient than six generations in the past.

Obtaining additional information on the admixture levels, timing and inheritance patterns can improve our understanding on the underlying factors favoring hybridization and its possible consequences, thus supporting the identification of the most appropriate conservation needs.

Consequently, management actions should be mainly aimed at reducing the high number of free-ranging cats within the current wildcat distribution, deserving particular attention to those areas where ecological barriers are not so strong to limit hybridization. Furthermore, priority management actions, such as captivation or sterilization, should be primarily addressed to recent generation hybrids, which carry significant portions of domestic genome ancestry, and eventually further extended toward more ancient backcrosses when they locally occur at high prevalence, thus increasing the probability of interbreeding and retaining domestic variants.

Future genome-wide scanning of a larger number of individuals from the whole European wildcat distribution range and the application of the optimized small marker panels in non-invasive genetic monitoring projects will contribute to (1) assess the hybrid frequencies and the current rates of domestic introgression in the wild populations, (2) provide information on the health status of wild-living individuals (through the analysis of genes related to illness, immune response, reproductive patterns or adaptation to specific ecological pressure), (3) identify areas with high conservational priority where try to limit the occurrence of hybridization and support appropriate local management practices.

## Materials and Methods

### Ethical statements

No ethics permit was required for this study, and no animal research ethics committee prospectively was needed to approve this research or grant a formal waiver of ethics approval.

### Sampling

DNA was extracted from blood or muscular tissue samples collected from 100 presumed European wildcats (WC), 46 domestic cats (DC) and 36 known or presumed WC *x* DC admixed (HY; Table 3). Samples were collected from a large part of the wildcat distribution range in Europe, including the five main genetic clusters identified by Mattucci *et al*.^27^: Iberian, Italian, Central European, Dinaric and Central Germany populations (Supplementary Fig. S1). All cats were previously analyzed with a few tens of microsatellites^20,21,27,37^.

**Table 3:** Origin and sample size of the genotyped domestic cats (*Felis silvestris catus*), European wildcats (*F*. *s*. *silvestris*) and their putative admixed cats.

Subspecies	Acronyms	Macro-populations	No.
Domestic cats	DC	Italy	14
		Dinaric Alps	4
		Central Europe	5
		Iberia	23
		**Sub-total**	46
Putative and known admixed cats	Hyb	Italy: captivity (known)	8
		Italy	16
		Dinaric Alps	3
		Central Europe	3
		Iberia	6
		**Sub-total**	36
European wildcats	WC	Italy	15
		Dinaric Alps	35
		Central Germany	10
		Central Europe	21
		Iberia	19
		**Sub-total**	100
		**Total (all cats)**	182

All cats were preliminarily assigned to their most likely subspecies/macro-population of origin by Bayesian clustering and admixture analyses performed in Mattucci *et al*.^27^.

The vast majority (97%) of the samples used in this study were collected from found-dead cats by specialized technician personnel for scientific purposes. The blood samples (n = 5) were collected with permission from owners from domestic cats by veterinarians during their routine health examinations. Additionally no anesthesia, euthanasia, or any kind of animal sacrifice was applied for this study and all blood samples were obtained aiming at minimizing the animal suffering. No ethics permit was required for this study, and no animal research ethics committee prospectively was needed to approve this research or grant a formal waiver of ethics approval.

### Quality-control of the DNA samples and SNP genotyping

Genomic DNA was extracted using the Qiagen DNAEasy Blood and Tissue kits (Qiagen Inc, Hilden, Germany) according to the manufacturer’s instructions, quantified using the Infinite200 PRO NanoQuant (Tecan System Inc, San Jose, USA) and visually-controlled for DNA degradation by standard 1.5% agarose gel electrophoresis. An initial panel of 182 samples, showing no DNA degradation and at least 50 ng/ul DNA, was genotyped using the Infinium iSelect 63k Cat DNA Array (Illumina Inc., San Diego, CA) including 62,897 SNP positions of which 4,240 were wildcat-specific^49^. Considering the alignment of the markers to *Felis_catus*_8.0^55^ (ICGSC; https://www.ncbi.nlm.nih.gov/assembly/GCF_000181335.2/), 704 SNPs (including 7 insertion/deletion makers) did not map to any chromosomes or anchored contigs and were excluded from the analyses. X-linked SNPs (n = 2,724 SNPs) were further excluded. The remaining 59,469 autosomal SNP genotypes were then filtered for individual missingness rates (GENO > 0.2), individual missing call rate (MIND > 0.2) and number of invariant SNPs in Plink^73^, resulting in a starting dataset of 146 samples genotyped at 57,302 SNPs (the 57k SNP panel set). This panel included 92% of the wildcat-specific variants^38^ (n = 3,885). The initial dataset was also pruned for Linkage Disequilibrium (LD), filtering for *r*^2^ > 0.5 in a 50-SNP sliding windows, shifted and recalculated every five SNPs. LD-filtered loci resulted in a dataset of 146 samples genotyped for 35,228 SNPs (the 35k LD-pruned SNP panel set). Based on the analysis undertaken, the most appropriate SNP panel set was utilized.

### Admixture analyses and assignment of the individual genotypes

Patterns of genetic differentiation among samples was explored by a preliminary non-model Principal Components Analysis^74^ (PCA) in the SNP&Variant Suite v.8.0.1 (SVS, Golden Helix Inc., Bozeman, MT) using the 57k SNP panel set. Each sample was then reassigned to its population of origin running the 35k LD-pruned SNP panel set in Admixture v.1.23^75^ assuming K values from 1 to 20. The most likely number of clusters was identified based on the lowest cross validation error^75^ and results were plotted in R v.3.5.0 (www.r-project.org, last accessed April 23, 2018). Individual ancestry components assessed with Admixture (at K = 2) were then used to select the reference wildcats, reference domestic cats and admixed individuals for all the subsequent analyses (see results).

The occurrence of admixture events in the European wildcat populations was formally tested on the 57k SNP panel set with the F3-statistics running the ThreePop program implemented in TreeMix v.1.12, using blocks of 20 adjacent SNPs to estimate standard errors, and Z-score values < −3 to significantly indicate admixture in the target population^53^.

The 57k SNP panel set was further used to infer local ancestry along individual chromosomes and to calculate genome-wide proportions of admixture, through a PCA-based approach implemented in the PCAdmix v.1.0^56^. Each chromosome was analyzed independently, running blocks of 20 consecutive, non-overlapping SNPs, and local-ancestry assignment was based on loadings from principal-component (PC) analysis on the two putative ancestral populations’ panels (the reference wildcats and the reference domestic cats). For each admixed individual, we then calculated the average genome-wide proportion of blocks assigned to each reference population.

The reliability of the selected reference populations to detect admixture signals and the absence of any possible ascertainment bias linked to the original SNP chip design based on the domestic cat variation were tested by reanalyzing 20 baseline wildcats and 20 baseline domestic cats, randomly chosen, as putative hybrids in PCAdmix.

### Time of admixture

We reconstructed chromosomal haplotypes in Shapeit v.2.837^76^ using the 57k SNP panel set with default parameter settings and considering domestic cat recombination maps^55^. The phased haplotypes were then used to estimate the average time of admixture events between the reference populations (domestic and wild cats) in Alder v.1.03^54^, which models the signature of decay in Linkage Disequilibrium (LD) between a pair of sites located on the same chromosome as the distance between these sites increases. The putative admixed individuals were first analyzed as a unique cohort and then grouped into cohorts representative of each European wildcat macro-population. Significant admixture events were assessed at P-values < 0.01 and then compared to those estimated using the number of ancestry switches inferred with PCAdmix with the formula developed by Johnson *et al*.^77^, and converted into years since sampling assuming a generation time of two years^78^.

### Local genome ancestry, gene search and gene ontology

The admixture mapping reconstructed by PCAdmix was finally used to adaptively search for introgressed alleles in the domestic *x* wildcat admixed individuals. We first selected the genome-wide regions showing an excess of domestic or wild cat contributions in the admixed individuals identified by Admixture. Chromosomal haplotype blocks of 20 SNPs were thus ranked according to their relative proportion of “domestic cat” or “wildcat” assignment detected by PCAdmix (corresponding to 100% domestic or 100% wild cat ancestry, respectively) in order to subsequently identify within them only the top and bottom 1% of the genome-wide frequency distribution that is expected to be enriched for genes bearing signature of positive selection after admixture^79^.

We additionally identified F_ST_ outlier SNPs at a significant P < 0.05 in BayeScan^80^ for evidence of selection signatures by comparing admixed individuals *versus* wildcats and admixed individuals *versus* domestic cats. The analysis was performed using default parameter values of 100,000 iterations after an initial burn-in of 50,000 steps, setting a maximum False Discovery Rate (FDR) = 0.05 (the allowed proportion of false positives), and a q value = 10% (the minimum FDR at which a locus may become significant). Outlier regions were obtained including 100 Kb on each side of the F_ST_ outlier SNPs detected by BayeScan, assuming an average LD in domestic cats of 96 Kb^81^.

Finally, we recovered the genes included in each domestic-like and wildcat-like outlier region obtained from both methods based on the Ensembl gene annotation 92 in Biomart^82^ (http://www.ensembl.org/biomart/martview/), and checked them for their possible enrichment for any Gene Ontology (GO), Biological Processes (BP) and Human Phenotypes (HP) categories available in G-profiler^83^. Enrichment was tested retaining those categories that were significant at P < 0.05 after Benjamini–Hockberg correction.

### Selection of informative autosomal SNPs for both ancestry detection and population structure

The 35k LD-pruned SNP panel set was examined to identify a reduced set of ancestry-informative SNPs (AIMs). We first identified the most divergent SNPs between domestic cat and wildcat genotypes (showing Admixture q_w_ = 1.000, and confirmed by PCAdmix, see Results) and ranked the SNPs for decreasing wild *x* domestic cat F_ST_ values in SVS and for informativeness of the assignment index (I_N_) in Infocalc^84^. The Spearman Rank correlation, *r*, was estimated among F_ST_ and I_N_ ranks and its significance was tested with a Student’s t test. Three panel sets of 192, 96, and 48 SNPs were finally selected for assignment procedures performed in Admixture and PCA analyses in SVS to estimate their power to clearly identify reference parental populations (wild and domestic cats) and their admixed individuals.

Subsequently, we identified the most informative SNPs to distinguish the main European wildcat macro-populations^27^, ranking the markers for decreasing macro-pop average F_ST_ values in SVS and selecting two reduced panel sets of 96 and 48 SNPs that were reanalyzed in a PCA plot.

Finally, all the reduced panel sets of markers were combined to develop the most affordable panel set informative for both admixture and genetic structure analyses.

## Supplementary information

Supplemetary Information

Supplemetary Table S1

## Data Availability

The majority of the data generated and analyzed during the current study are presented within the published article or in Supplementary information files. The raw data are available from the corresponding author on reasonable request.
