# Supplementary material for: Genomic approaches to identify hybrids and estimate admixture times in European wildcat populations
Source: Sci Rep. 2019 Aug 12;9:11612. doi: 10.1038/s41598-019-48002-w (PMC6691104; doi:10.1038/s41598-019-48002-w)
Supplement: Supplementary file 1 — Supplemetary Information [file 41598_2019_48002_MOESM1_ESM.docx]

**Genomic approaches to identify hybrids and estimate admixture times in European wildcat populations**

**Federica Mattucci**^1^***, Marco Galaverni**^2^**, Leslie A. Lyons**^3^**, Paulo C. Alves**^4,5,6^**, Ettore Randi^7,8^, Edoardo Velli**^1^**, Luca Pagani**^9,10^ **and Romolo Caniglia**^1^

^1^ Area per la Genetica della Conservazione (BIO-CGE), Istituto Superiore per la Protezione e la Ricerca Ambientale (ISPRA), Ozzano dell’Emilia, Italy

^2^ Area Conservazione, WWF Italia, Rome, Italy

^3^ Department of Veterinary Medicine and Surgery, College of Veterinary Medicine, University of Missouri, Columbia, USA

^4^ Centro de Investigação em Biodiversidade e Recursos Genéticos (CIBIO), InBio - Laboratório Associado, Campus Agrário de Vairão, Vairão, Portugal

^5^ Departamento de Biologia, Faculdade de Ciências, Universidade do Porto, Porto, Portugal

^6^ Wildlife Biology Program, Department of Ecosystem and Conservation Sciences, University of Montana, Missoula, USA

^7^ Department of Biological, Geological and Environmental Sciences, University of Bologna, Bologna, Italy

^8^ Department of Chemistry and Bioscience, Faculty of Engineering and Science, University of Aalborg, Aalborg, Denmark

^9^ Dipartimento di Biologia, Università degli Studi di Padova, Padua, Italy

^10^ Estonian Biocentre, Institute of Genomics, University of Tartu, Tartu, Estonia

*Correspondence and requests for materials should be addressed to: federica.mattucci@isprambiente.it

**Supplementary Information**

**Supplementary text S1**

**Discussion on the regions with excess of ancestry or introgression detected from admixture mapping**

Across the genomes of the analyzed admixed individuals, a total of 138 wildcat-like regions were identified, including 577 significantly enriched genes for Cellular Component (CC) categories implicated in several biological and cognitive processes related to communication and elusive behaviors (Table 1 and Supplementary Table 1 c-d). In particular, they included genes playing important roles in memory performance (*ADCY8^1^, CUX2*^2^) and sociability, such as *DYNLRB2*^3^ and *MRPS18A*^4^*,* both up-regulated in high fearful animals; *MAD2L1BP*^5^*,* which was likely over-expressed in male individuals following territorial intrusions within sticklebacks populations; *ATP8A1*^6^*,* responsible for aberrant social behavior related to feeding efficiency and poor maternal attitude; *ADCY7*^7^*,* responsible for altered emotional responsiveness in mice; and two genes (*SPTLC3*^8^ and *SLC6A4*^9^), significantly associated with aggression, which encode the serotonin transporter density directly correlated to the hostility behavior. Additionally, a set of wildcat-like genes included in enriched CC categories was described to be related to brain development: *DCTN2*^10^, responsible to the synapse formation, and *KIT*^11^, which was previously described for regulating the white coats patterns in various mammalian species^12^ and has been considered a hallmark of cat domestication since it regulates the spotting pigmentation phenotype of several white-spotted cats^13^. A variety of genes belonging to enriched CC categories was further described to be associated with development processes and key morphological features, such as *EDN1* and *KIF2B,* responsible for the regulation of pressure^14^ and coagulation of blood^15^; *BHMT*^16^, which codes for specific enzymes involved in energy balance and nutrient partitioning; and various genes implicated in milk yielding (*ACP6*^17^, *ARHGEF4*^18^, *MTX3*^18^), body size (*NPR2*^19^) and coat-hair features (*PIK3R4*^20^*, STX17*^21^). Interestingly, we found a few other enriched CC genes described to be correlated with fertility through the regulation of spermatogenesis (*MORN3*^22^, *NKD1*^23^), with the maintenance of pregnancy (*MCL1*^24^*, LNPEP*^25^), and with the likelihood of survival (*RNPEP*^26^).

Moreover we disclosed a series of wildcat-excess genes included in enriched CC categories previously described in the literature to be linked to a number of common disorders, some of which specifically found in felids, such as the congenital or juvenile primary cataracts (*BFSP2*^27^), the feline spinal muscular atrophy (*LIX1*^28^), the Hypertrophic Cardiomyopathy Mutations (*MYL2*^29^), in addition to a gene (*MYO1A*^30^) responsible for the hearing process in wild and farmed canids, which can cause hearing disorders or hypoacusis, as well as substantial behavioral changes in dogs (ranging from timidity to aggressive behaviour^30^).

Moreover, 138 segments with high frequency of domestic alleles were identified, containing 902 annotated genes, 39 of which were included in significantly enriched Human Phenotype (HP) and Molecular Function (MF) categories, mostly related to cell adhesion molecular binding (Supplementary Table S1 a-b). Interestingly, among them, four domestic-like genes were described in the literature as associated with cognition and physiological adaptation: *PRC5A*^3^, previously described in fear-of-human selection lines of the red junglefowl; *CAPZA1*^31^, which is related to the biological immune system response to exercised-induced stress in horses (Table 1).

Many functional enriched GO genes appeared to be neutrally inherited in admixed individuals from both wild and domestic cats. Both wild-like and domestic-like regions hosted genes belonging to enriched GO and involved in lipid metabolism (*PLPP3*^32^*, ALDH2*^33^), muscle development and energy metabolism (*MYLPF*^34^), which may act as a regulator of the muscle respiratory chain activity^35^ and be directly linked to a diet-induced obesity/insulin resistance (*COX6A2*^36^). Their enrichment in the admixed genomes might be considered a signature of adaptation for accommodating the hyper carnivorous diet of felids^37^, through the regulation of fatty acids metabolism^38^, which contributed to differ them from the other carnivores^37^.

Moreover, wild and domestic regions included several enriched GO genes involved in tumor suppression (*PHLDB2*^39^, *TRIM29*^40^, *PTPRT*^41^), most of which are further related to stress responses (*BIN1*^42^), cell cycle regulation (*CUL9*^43^), skin development (*GSDMC*^44^), internal nucleolar structure and cell growth rates (*NOL7*^45^). Additionally, we identified some enriched GO genes known to be involved in immune functions, such as those coding for modulating the infection of feline leukemia virus (FeLV) (*TRIM25*^46^) and the influenza A viruses severity (*ZC3HAV1*^47^), those responsible for species-specific responses to ASFV (*NFKBIE*^48^) and H5N1 virus infections (*SLC16A1*^49^), those accountable for positive response to a wide variety of cell stresses and DNA repair (*CMPK1*^50^, *DDIT3*^51^, *CCDC92*^52^) and a gene (*BCL9*) associated to heart diseases^53^.

Overall, further disentangling the role of these genes in the adaptation of admixed individuals can shed light on their contribution to the fitness of individuals and on the probability of introgressions of specific domestic alleles through generations, in order to prioritize the best intervention strategies for the conservation of wildcats.

**References**

1. De Quervain, D. J. & Papassotiropoulos, A. Identification of a genetic cluster influencing memory performance and hippocampal activity in humans. *PLoS One* **103**, 4270-4274 (2006).

2. Cubelos, B. *et al.* Regulate dendritic branching, spine morphology, and synapses of the upper layer neurons of the cortex. *Neuron* **66**, 523-535 (2010).

3. Bélteky, J., Agnvall, B., Johnsson, M., Wright, D. & Jensen, P. Domestication and tameness: brain gene expression in red junglefowl selected for less fear of humans suggests effects on reproduction and immunology. *Royal. Soc. Open Sci*. **3**, 160033, DOI:10.1098/rsos.160033(2016).

4. Bélteky, J., Agnvall, B. & Jensen, P. Gene expression of behaviorally relevant genes in the cerebral hemisphere changes after selection for tameness in red junglefowl. *PLoS One* **12**, e0177004 (2017).

5. Greenwood, A. K. & Peichel, C. L. Social regulation of gene expression in threespine sticklebacks. *PLoS One* **10**(9), e0137726, DOI:10.1371/journal.pone.0137726 (2015).

6. Kerr, D. J. *et al.* Aberrant hippocampal Atp8a1 levels are associated with altered synaptic strength , electrical activity , and autistic-like behavior. *BBA - Mol. Basis Dis.* **1862**, 1755-1765 (2016).

7. Joeyen-Waldorf, J. *et al.* Adenylate Cyclase 7 is implicated in the biology of depression and modulation of affective neural circuitry. *BPS* **71**, 627-632 (2012).

8. Pavlov, K. A., Chistiakov, D. A. & Chekhonin, V. P. Genetic determinants of aggression and impulsivity in humans. *J. Appl. Genet.* **53**(1), 61-82, DOI:10.1007/s13353-011-0069-6 (2012).

9. Van den Berg, L. *et al.* Evaluation of the serotonergic genes htr1A, htr1B, htr2A, and slc6A4 in aggressive behavior of Golden Retriever dogs. *Behav. Genet.* **38**, 55-66 (2008).

10. Uetake, Y., Terada, Y., Matuliene, J. & Kuriyama, R. Interaction of Cep135 With a p50 dynactin subunit in mammalian centrosomes. *Cell. Motil. Cytoskeleton* **66**, 53-66 (2004).

11. Fleischman, R. A., David, L., Stastny, V. & Zneimer, S. Deletion of the c-kit protooncogene the human developmental defect piebald trait. *Proc. Natl. Acad. Sci. USA* **88**, 10885-10889 (1991).

12. Pulos, W. L. & Hutt, F. B. Lethal Dominant White in Horses. *J. Hered.* **60**, 59-63 (1969).

13. David, V. A. *et al.* Endogenous retrovirus insertion in the KIT oncogene determines white and white spotting in domestic cats. *G3* **4**, 1881 LP-1891 (2014).

14. Kurihara, Y. *et al.* Elevated blood pressure and craniofacial abnormalities in mice deficient in endothelin-1. *Nature* **368**, 703 (1994).

15. Ai, H. *et al.* Adaptation and possible ancient interspecies introgression in pigs identified by whole-genome sequencing. *Nat. Genet.***47**, 217 (2015).

16. Ponsuksili, S., Murani, E. & Schellander, K. Identification of functional candidate genes for body composition by expression analyses and evidencing impact by association analysis and mapping. [*Biochim. Biophys. Acta.*](https://www.ncbi.nlm.nih.gov/pubmed/16005530) **1730**, 31-40 (2005).

17. Moioli, B., Andrea, M. D. & Pilla, F. Candidate genes affecting sheep and goat milk quality. *Small Rumin. Res.* **68**, 179-192 (2007).

18. Chen, Z., Yao, Y., Ma, P., Wang, Q. & Pan, Y. Haplotype-based genome-wide association study identifies loci and candidate genes for milk yield in Holsteins. *PLoS One* **13**(2), e0192695, DOI: 10.1371/journal.pone.0192695.

19. Chase, K., Jones, P., Martin, A., Ostrander, E. A. & Lark, K. G. Genetic mapping of fixed phenotypes: disease frequency as a breed characteristic. *J. Hered.* **100**, S37-S41 (2009).

20. Wang, Z. *et al.* Genome-Wide Association study for wool production traits in a Chinese Merino sheep population. *PLoS One* **9**(9), e107101, DOI:10.1371/journal.pone.0107101 (2014).

21. Pielberg, R. G. *et al.* A cis-acting regulatory mutation causes premature hair graying and susceptibility to melanoma in the horse. *Nat. Genet.* **40**, 1004 (2008).

22. Zhang, L. *et al.*Characterization of membrane occupation and recognition nexus repeat containing 3, meiosis expressed gene 1 binding partner , in mouse male germ cells. *Asian J. Androl.* **17**(1), 86-93, DOI: 10.4103/1008-682X.138186 (2015).

23. Li, Q., Ishikawa, T., Miyoshi, H., Oshima, M. & Taketo, M. M. A targeted mutation of Nkd1 impairs mouse spermatogenesi. *J. Biol. Chem.* **280**(4), 2831-2839, DOI:10.1074/jbc.m405680200 (2005).

24. Boumela, I. *et al.* Involvement of BCL2 family members in the regulation of human oocyte and early embryo survival and death: gene expression and beyond. *Reproduction* **141**, 549-561 (2011).

25. Kim, J. *et al.* Sequence variants in oxytocin pathway genes and preterm birth: a candidate gene association study. *BMC Med.Genet.* **14**(1), DOI:10.1186/1471-2350-14-77 (2013).

26. Cawthon, M. C., Kerber, R. A., Hasstdet. S. J. & O'Brien E. Methods and kits for d etermining biological age and longevity based on gene expression profiles. *U.S. Patent Application* **13**(28), 910 (2011).

27. Philipp, U. Steinmetz, A. & Distl, O. Development of feline microsatellites and SNPs for evaluating primary cataract candidate genes as cause for cataract in Angolan lions (*Panthera leo bleyenberghi*). *J. Hered.* **101**, 633-638 (2010).

28. Fyfe, J. C. *et al.* An similar to 140-kb deletion associated with feline spinal muscular atrophy implies an essential LIX1 function for motor neuron survival. *Genome Res.* **16**, 1084-1090 (2006).

29. Flavigny, J. *et al.* Identification of two novel mutations in the ventricular regulatory myosin light chain gene (MYL2) associated with familial and classical forms of hypertrophic cardiomyopathy. *J. Mol. Med.* **76**(3-4), 208-214 (1998).

30. Andrzej, J., Magdalena, G. & Beata, H. SNP genetic diversity within a fragment of the gene myo15a responsible for the hearing process in a population of farmed and free-living animals of the canidae family. *Acta Vet.* **64**, 358-366 (2014).

31. Park, W. *et al.* Investigation of de novo unique differentially expressed genes related to evolution in exercise response during domestication in thoroughbred race horses. *PLos One* **9**(3), e91418, DOI:10.1371/journal.pone.0091418 (2014).

32. Lan, D. *et al.* Genetic diversity, molecular phylogeny, and selection evidence of Jinchuan yak revealed by whole-genome resequencing. *G3* **8**, 945-952 (2018).

33. Dunner, S. *et al.* Genes involved in muscle lipid composition in 15 European *Bos taurus* breeds. *Animal Genet.* **44**(5), 493-501, DOI:10.1111/age.12044 (2013).

34. Ghosh, M. *et al.* An integrated in silico approach for functional and structural impact of non- synonymous SNPs in the MYH1 gene in Jeju Native Pigs. *BMC Genet.* **17**(1), DOI:10.1186/s12863-016-0341-1 (2016).

35. Li, Y. *et al.* Full Paper A survey of transcriptome complexity in Sus scrofa using single-molecule long-read sequencing. *DNA Res.* **25**(4), 421-437, DOI:10.1093/dnares/dsy014 (2018).

36. Quintens, R. *et al.* Mice deficient in the respiratory chain gene Cox6a2 are protected against high-fat diet-induced obesity and insulin resistance. *PLoS One* **8**(2), e56719, DOI:10.1371/journal.pone.0056719 (2013).

37. Montague, M. J. *et al.* Comparative analysis of the domestic cat genome reveals genetic signatures underlying feline biology and domestication. *Proc. Natl. Acad. Sci.* **111**, 1-6 (2014).

38. Ibeagha-awemu, E. M., Peters, S. O., Akwanji, K. A., Imumorin, I. G. & Zhao, X. High density genome wide genotyping-by-sequencing and association identifies common and low frequency SNPs, and novel candidate genes influencing cow milk traits. *Sci. Rep.* **6**(1), DOI:10.1038/srep31109 (2016).

39. Chen, G., Zhou, T., Li, Y., Yu, Z. & Sun, L. p53 target miR-29c-3p suppresses colon cancer cell invasion and migration through inhibition of PHLDB2. *Biochem. Biophys. Res. Commun.* **487**(1), 90-95, DOI:10.1016/j.bbrc.2017.04.023 (2017).

40. Ai, L., Kim, W. & Alpay, M. TRIM29 Suppresses TWIST1 and invasive breast cancer Behavior. *Cancer Res.* **74**(17), 4875-4887, DOI:10.1158/0008-5472.CAN-13-3579 (2014).

41. Bermingham, M. L. *et al.* Genome-wide association study identifies novel loci associated with resistance to bovine tuberculosis. *Heredity* **112**(5), 543-551, DOI:10.1038/hdy.2013.137 (2014).

42. Muller, A. J. *et al.* Targeted disruption of the murine Bin1 / Amphiphysin II gene does not disable endocytosis but results in embryonic cardiomyopathy with aberrant myofibril formation. *Mol. Cell. Biol*. **23**(12), 4295-4306 (2003).

43. Sell-Kubiak, E. *et al.* Genome-wide association study reveals novel loci for litter size and its variability in a Large White pig population. *BMC Genomics* **16,** 1049 (2015).

44. Xiaolong, W. *Identification and Characterization of Candidate Genes for Complex Traits in Cattle.* PhD Thesis. Technische Universität München (2013).

45. Hasina, R. *et al.* NOL7 is a nucleolar candidate tumor suppressor gene in cervical cancer that modulates the angiogenic phenotype. *Oncogene* **25**(4), 588-598, DOI:10.1038/sj.onc.1209070 (2006).

46. Koba, R., Oguma, K. & Sentsui, H. Overexpression of feline tripartite motif-containing 25 interferes with the late stage of feline leukemia virus replication. *Virus Res.* **204**, 88-94 (2015).

47. Powell, J. D. & Waters, K. M. Influenza-omics and the host response : recent advances and future prospects. *Pathogens* **6**(2), 25, DOI:10.3390/pathogens6020025 (2017).

48. Palgrave, C. J. *et al.* Species-Specific variation in RELA underlies differences in NF- B activity: a potential role in african swine fever pathogenesis. *J. Virol.* **85**(12), 6008-6014, DOI:10.1128/jvi.00331-11 (2011).

49. Ranaware, P. B., Mishra, A. & Vijayakumar, P. Genome wide host gene expression analysis in chicken lungs infected with avian influenza viruses. *PLoS One* **11**(4), DOI:10.1371/journal.pone.0153671 (2016).

50. Tsao, N., Lee, M. H., Zhang, W., Cheng, Y. C. & Chang, Z. F. The contribution of CMP kinase to the efficiency of DNA repair. *Cell Cycle* **14,** 354-363 (2015).

51. Vattem, K. M. & Wek, R. C. Reinitiation involving upstream ORFs regulates ATF4 mRNA translation in mammalian cells. *Proc. Natl. Acad. Sci. USA* **101**, 11269-11274, DOI:10.1073/pnas.0400541101 (2004).

52. Haas, A. V & McDonnel, M. E. Pathogenesis of Cardiovascular Disease in Diabetes. *Endocrinol. Metab. Clin. NA.* **47**, 51-63 (2018).

53. Cantù, C. *et al.* Mutations in Bcl9 and Pygo genes cause congenital heart defects by tissue-specific perturbation of Wnt/β-catenin signaling. *Genes & Development* DOI:10.1101/gad.315531.118 (2018).

**Supplementary Table** (Separate file: Mattucci et al_Supplementary Table S1.xlsx)

**Supplementary Table S1.** a) Domestic excess genes surrounding the outlier domestic-like SNPs identified from PCAdmix. b) Enrichment in gene ontology (GO) categories of the domestic excess genes surrounding the outlier domestic-like SNPs identified from PCAdmix. c) Wildcat excess genes surrounding the outlier wildcat-like SNPs identified from PCAdmix. d) Enrichment in gene ontology (GO) categories of the wildcat excess genes surrounding the outlier wildcat-like SNPs identified from PCAdmix.

**Supplementary Figures**


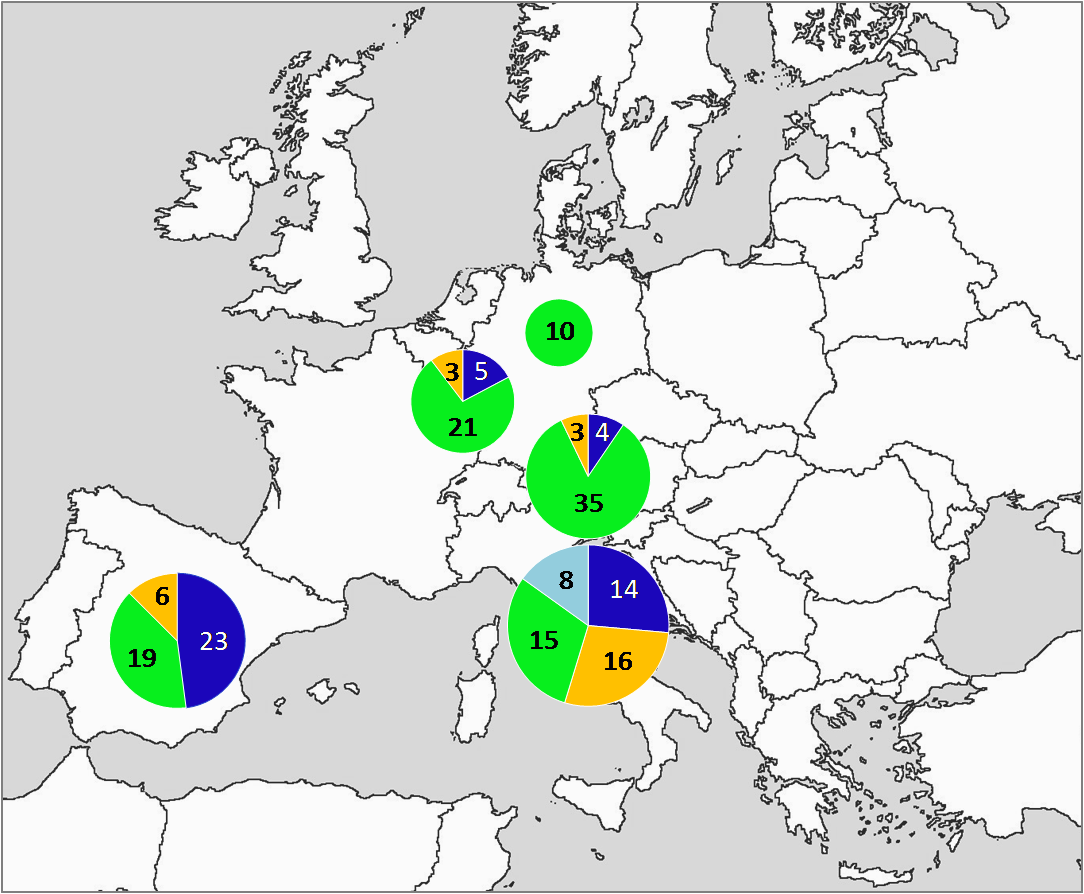


**Supplementary Figure S1.** Approximate sampling locations of cats collected across Europe**.** Putative domestic, European wild and admixed cat samples are numbered and represented with different colors (blue, green and orange, respectively) in circles whose size is scaled according to the samples abundance. Italian known hybrids from captivity are showed in light blue.

**

**

**Supplementary Figure S2.** Cross-validation error plot obtained from Admixture run on the 35k LD-pruned SNP panel set, with K from 1 to 20.

**
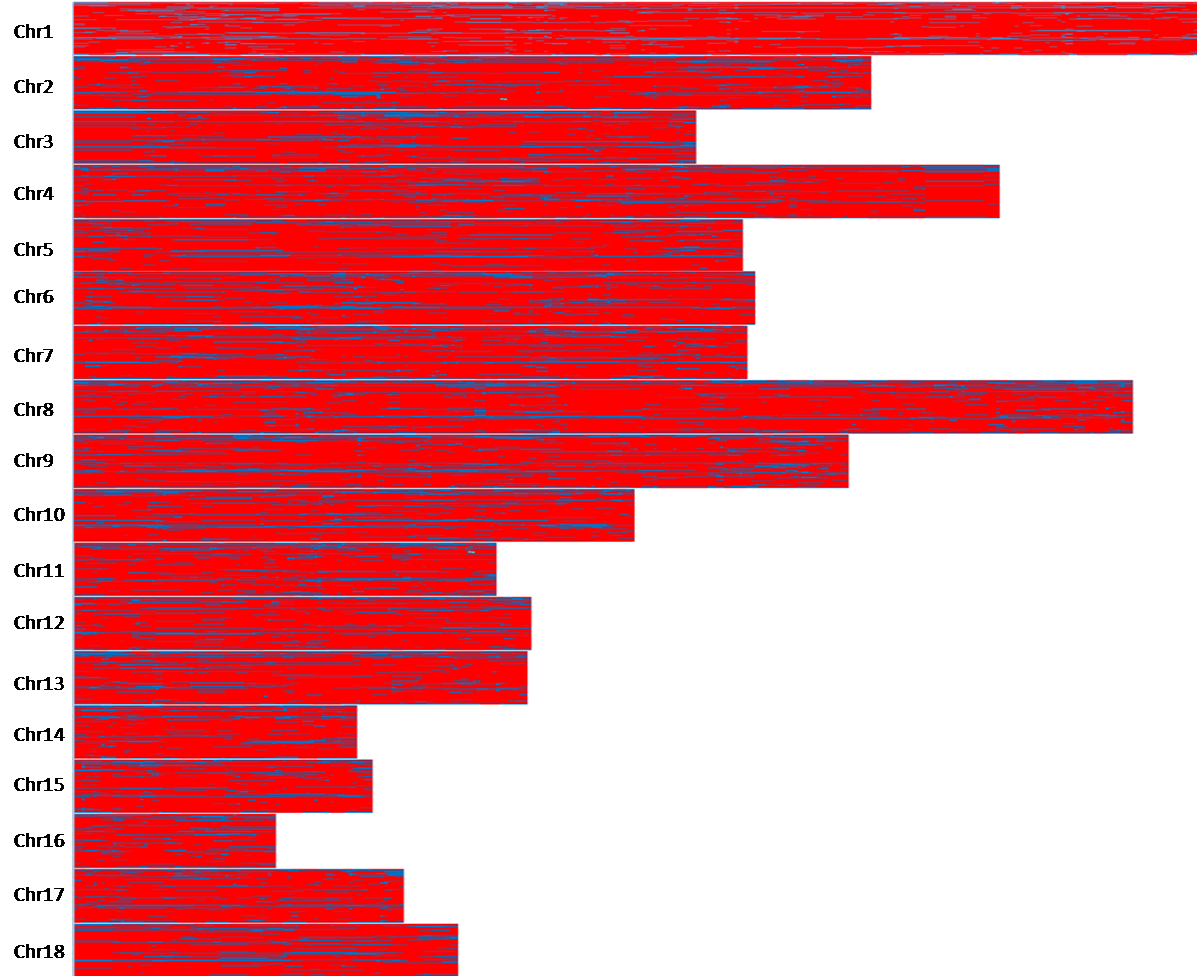
**

**Supplementary Figure S3.** Results of local ancestry block analysis in PCAdmix for 18 chromosomes. Domestic cat ancestry is marked in blue and wildcat ancestry in red. Each row represents one admixed individual (n = 45) detected with Admixture assignment.


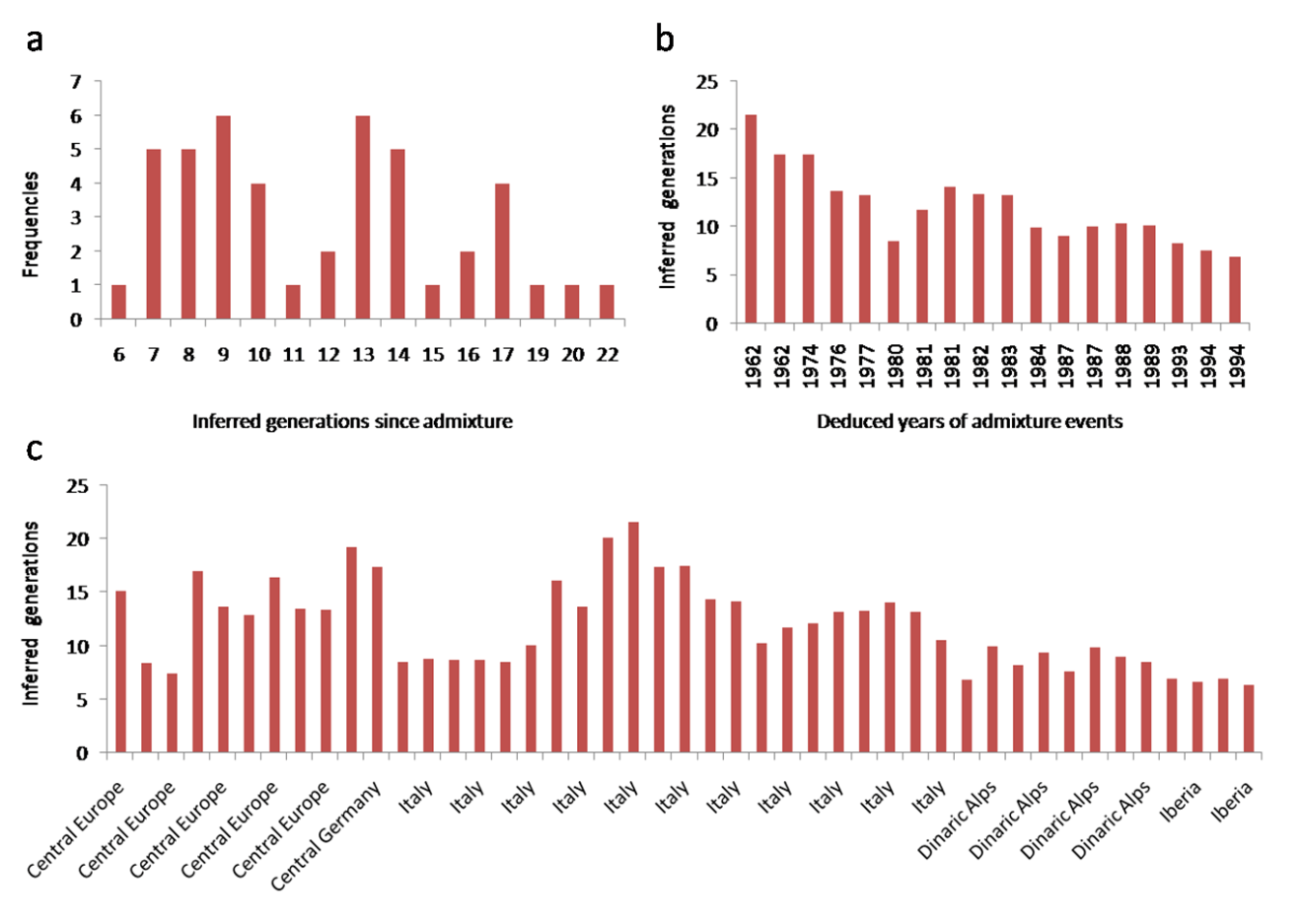


**Supplementary Figure S4.** Inferred number of generations since admixture in the European wild *x* domestic cat admixed individuals (n = 45) deduced from the number of switches from wild to domestic haplotype blocks assigned by PCAdmix (a) and corresponding temporal distribution of the admixture events since sampling assuming a generation time of two years (b), and spatial distribution of the admixture events (c).


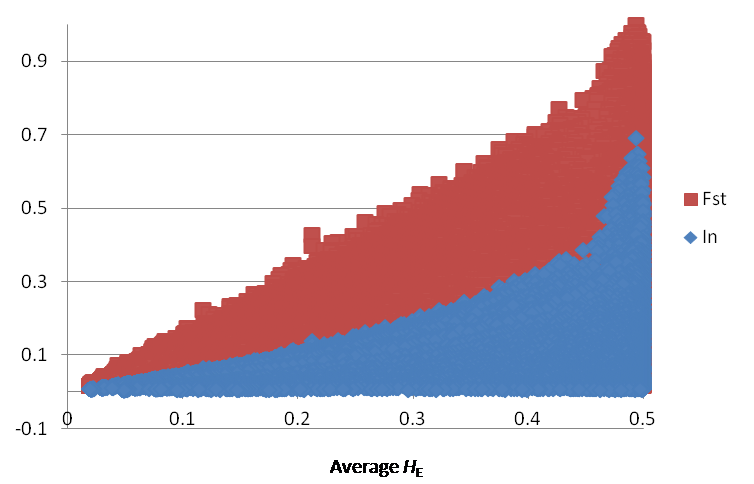


**Supplementary Figure S5.** Biplots of correlation among the average heterozigosity (H_E_) and the F_ST_ and the informativeness for assignment (I_N_) index for domestic (n = 44) and wild cats (n = 57) samples. Values were calculated for the 35k LD-pruned SNP panel set.

**
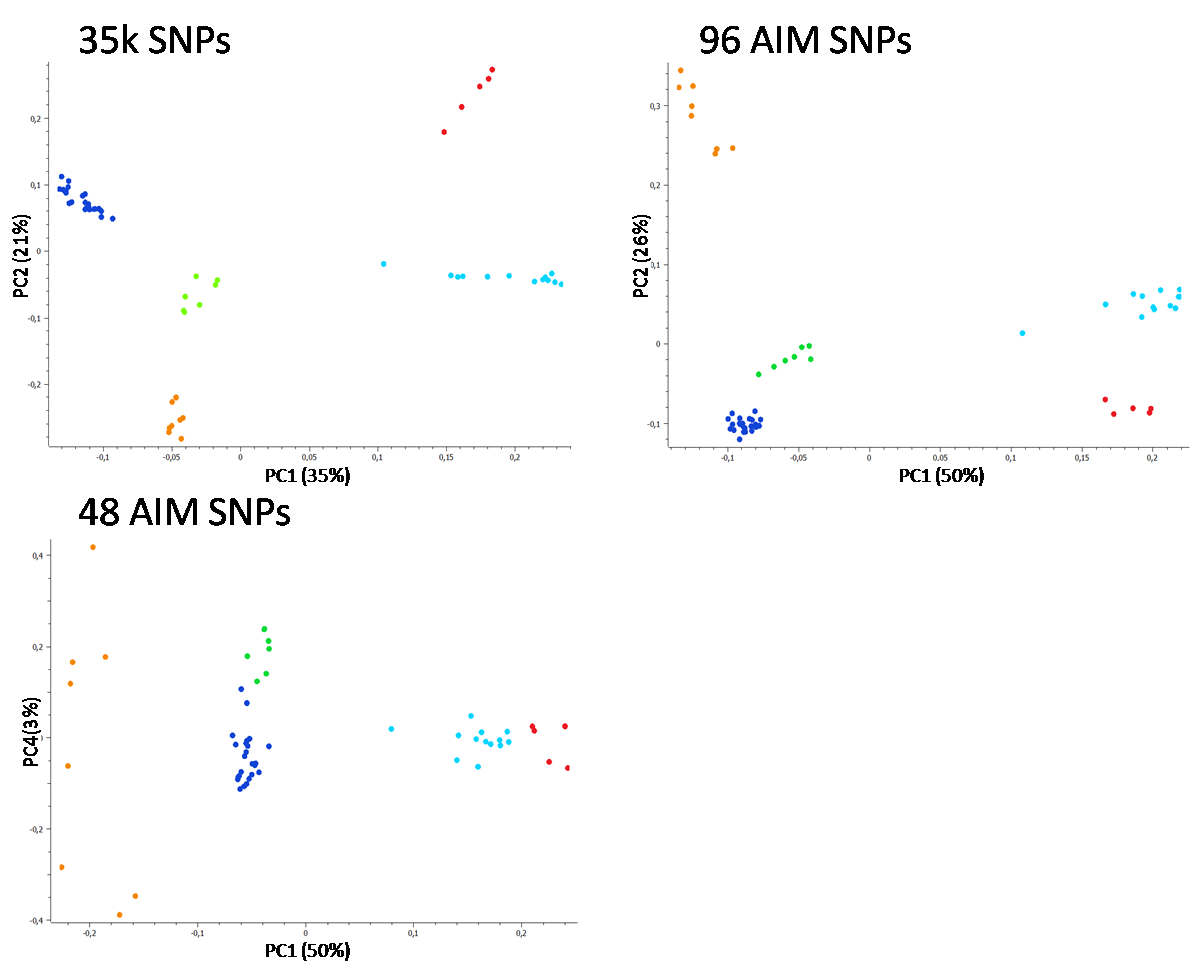
**

**Supplementary Figure S6**. Principal component analysis (PCA) computed in SVS on the 35k LD-pruned SNP panel set, the reduced 96 and 48 SNP panel sets showing the highest F_ST_ values among the five biogeographic macro-populations already identified in Europe through STR Bayesian analyses. The plots show the clustering patterns of the European wildcats Dinaric (in blue), Central European (in green), Central Germany (in orange), Iberian (in light blue) and Italian (in red) populations. Individuals (dots) are plotted within the orthogonal space defined by the first two PCA eigenvalues (inserts) and by the first PC1 *versus* the fourth PC4 (axes are not to scale).
